# Supplementary material for: Increased Potential of Bone Formation with the Intravenous Injection of a Parathyroid Hormone-Related Protein Minicircle DNA Vector
Source: Int J Mol Sci. 2021 Aug 23;22(16):9069. doi: 10.3390/ijms22169069 (PMC8396456; doi:10.3390/ijms22169069)

Supplementary Figure S1. Expression of potential false-positive signals from RT-PCR as no controls (no Rtase) performed for mcPTHrP 1-34+107-139 vector contamination via gel imaging. A. PTHrP gene expression of potential false-positive signals from qPCR for mc PTHrP 1-34+107-139 vector contamination via gel image. B. GAPDH gene expression for mc PTHrP 1-34+107-139 vector via gel image. Left line is no RT (no controls, no Rtase) and Right line is RT (with Rtase). (HEK 293T n=2, Mock n=2 and mcPTHrP 1-34+107-139 n=2).

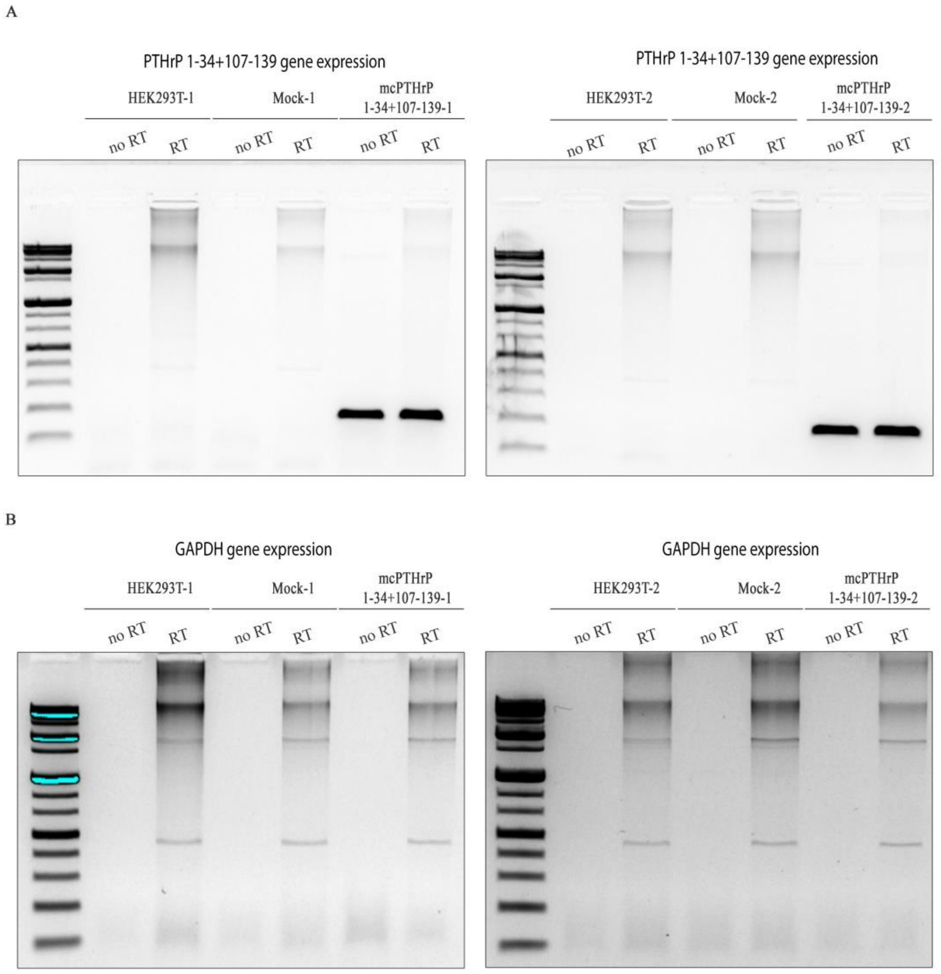

Supplement: Supplementary file 1 [file ijms-22-09069-s001.zip › Supplementary Figure S1.pdf]
