# Supplementary material for: Increased Potential of Bone Formation with the Intravenous Injection of a Parathyroid Hormone-Related Protein Minicircle DNA Vector
Source: Int J Mol Sci. 2021 Aug 23;22(16):9069. doi: 10.3390/ijms22169069 (PMC8396456; doi:10.3390/ijms22169069)

Supplementary Figure S2. Expression of potential false-positive signals from RT-PCR as no controls (no Rtase) performed for mc PTHrP 1-34+107-139 vector contamination *in vivo* via gel imaging. A. Expression of potential false-positive signals of mc PTHrP 1-34+107-139 in the spleen tissue. B. Results of potential false-positive signals of mc PTHrP 1-34+107-139 in the kidney tissue. C. Results of potential false-positive signals of mc PTHrP 1-34+107-139 in the liver tissue. Left line is no RT (no controls, no Rtase) and Right line is RT (with Rtase).

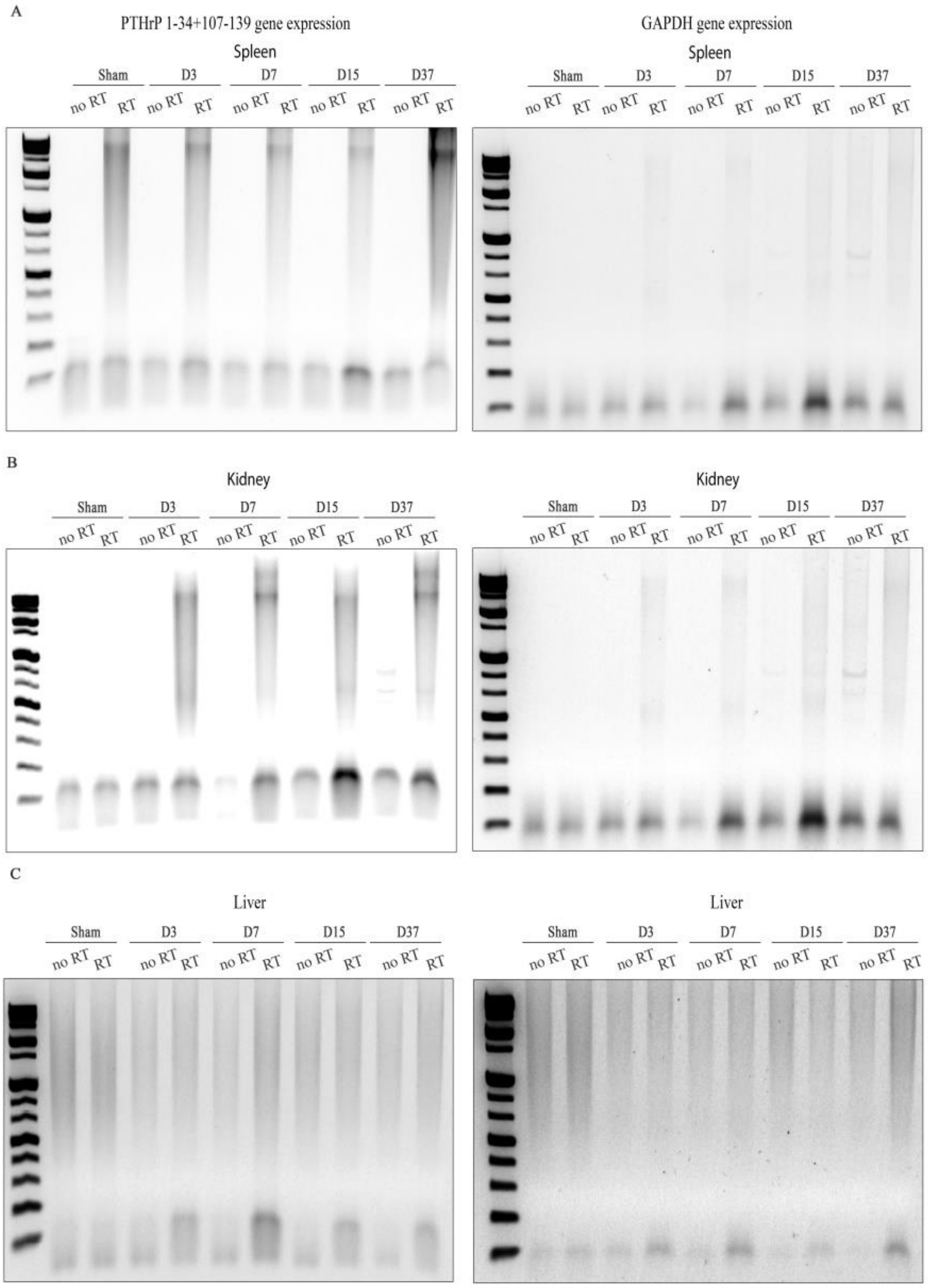

Supplement: Supplementary file 1 [file ijms-22-09069-s001.zip › Supplementary Figure S2.pdf]
