# Supplementary material for: Increased Potential of Bone Formation with the Intravenous Injection of a Parathyroid Hormone-Related Protein Minicircle DNA Vector
Source: Int J Mol Sci. 2021 Aug 23;22(16):9069. doi: 10.3390/ijms22169069 (PMC8396456; doi:10.3390/ijms22169069)

Supplementary Figure S3. mcPTHrP 1-34+107-139 synthesizing MSCs via microporation using the Neon transfection system. (A) Scheme of the generation process of mcPTHrP 1-34+107-139 MSCs (eMSCs). To confirm the transfection efficacy of mcPTHrP 1-34+107-139 MSCs via microporation using the Neon transfection system. Several conditions were tested for the microporation of mcPTHrP 1-34+107-139 vector transfection.

A

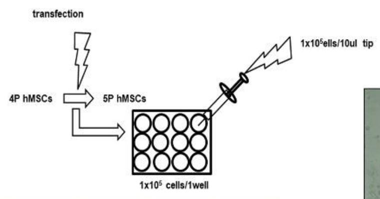

Sample no	Pulse voltage	Pulse width	Pulse no
1	1400	20	1
2	1600	20	1
3	1100	30	1
4	1300	30	1
5	1000	40	1
6	1200	40	1
7	1200	20	2
8	1400	20	2
9	950	30	2
10	1150	30	2
11	1400	10	3
12	1600	10	3

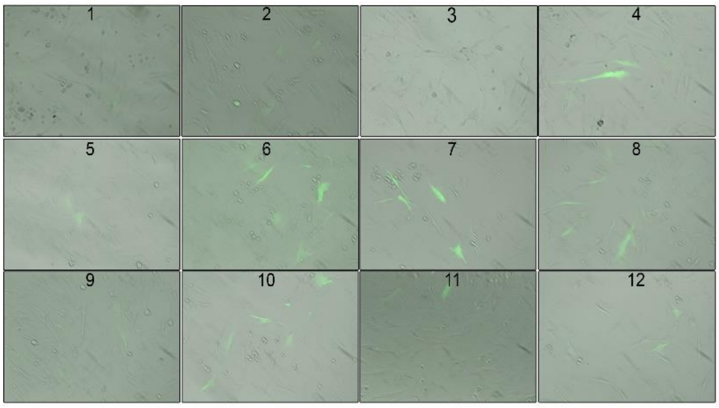

Supplement: Supplementary file 1 [file ijms-22-09069-s001.zip › Supplementary Figure S3.pdf]
