# Supplementary material for: Increased Potential of Bone Formation with the Intravenous Injection of a Parathyroid Hormone-Related Protein Minicircle DNA Vector
Source: Int J Mol Sci. 2021 Aug 23;22(16):9069. doi: 10.3390/ijms22169069 (PMC8396456; doi:10.3390/ijms22169069)
Supplement: Supplementary file 1 [file ijms-22-09069-s001.zip › Supplementary Table S1.pdf]

Supplementary Table S1. The sequence of PTHrP 1-34+107-139 inserts. The red text indicates the stop codon.

**PTHrP 1-34+107-139 cDNA sequence**
Gccaccatgggatggagctatatcatcctctttttggtggccacagcggccgatgtccactcggctgtgtctgaacatcagctcctccatgaca aggggaagtccatccaagatttacggcgacgattcttccttcaccatctgatcgcagaaatccacacagctactcgctctgcctggttagactc tggagtgactgggagtgggctagaaggggaccacctgtctgacacctccacaacgtcgctggagctcgattcacggtag
**PTHrP 1-34+107-139 amino acid peptide sequence**
ATMGWSYIILFLVATAADVHSAVSEHQLLHDKGKSIQDLRRRFFLHHLIAEIHTATRSAWLDSGVTGSGLEGDHLS DTSTTSLELDSR*
